# Selection of Policy Instruments on Integrated Care in China: Based on Documents Content Analysis

**DOI:** 10.3390/ijerph17072327

**Published:** 2020-03-30

**Authors:** Xin Yue, Kaining Mu, Lihang Liu

**Affiliations:** 1 School of Public Administration, Central South University, Changsha 410083, China; yuexin1115@csu.edu.cn; 2 School of Nursing, Xi’an Jiaotong University, Xi’an 710061, China; txm287317690@stu.xjtu.edu.cn

**Keywords:** policy instruments, integrated care, stakeholders, health service supply chains, China, article, yet reasonably common within the subject discipline

## Abstract

Facing the aggravating trend of an aging population and a fragmented medical service delivery system, the Chinese Central Government has introduced a series of policies to promote the development of integrated care against the background of the “Healthy China Strategy”. The achievement of integrated care depends on the choice of policy instruments. However, few studies have focused on how policy instruments promote the practice of integrated care in China. This article aims to obtain a deeper understanding of the use of policy instruments in the development of integrated care in China. Policy documents are the carriers of policy instruments. National-level integrated care policy documents from 2009 to 2019 were selected. Using the qualitative document analysis method, this paper conducts an analysis of integrated care policy instruments. In order to comprehensively view the integrated care policy instruments, a three-dimensional analytical framework consisting of the policy instruments dimension, stakeholders dimension, and health service supply chains dimension is proposed. The results are as follows. (1) From the perspective of policy instruments, the integrated care policy has adopted supply-side policy instruments, demand-side policy instruments, and environmental policy instruments. Among the three types of policy instruments, environmental policy instruments are used most frequently, supply-side policies are preferred, while demand-side policy instruments are relatively inadequate. (2) As for the stakeholders dimension, the central policy instruments focus on the health service providers, while less attention is paid to the health service demanders. (3) In terms of health service supply chains, the number of policy instruments used in the prevention stage is the highest, followed by the treatment stage, whereas less attention paid to the rehabilitation stage. Finally, suggestions were made for the development of integrated care by better perfecting policy instruments.

## 1. Introduction 

Enhancing healthcare service efficiency is the common pursuit of all countries. However, incoordination between health services providers has become a key factor that lowers the efficiency of health services. The incoordination care has led to wasted resources, medical errors, an inefficient use of medical resources, and depressing the satisfaction of the patient [1]. Increasing incoordination issues have compelled many countries to seek changes in the existing health service delivery system and explore new health service models to meet the people’s health services demands. As a result, integrated care has been brought onto the health reform agenda of many countries. The conception of “integrated care” was put forward at the end of the 20^th^ century [2,3]. It is based on the idea of providing one-stop health services to meet all the needs of people. A number of studies show that integrated care can also improve patient satisfaction, enhance efficiency, and promote quality [4,5,6]. At the beginning of the 21^st^ century, the World Health Organization (WHO) has advocated that countries should promote the development of integrated care and enhance the coordination ability of health service supply. At present, the transition from uncoordinated healthcare systems to integrated healthcare systems is a relatively popular trend in many countries [7,8,9,10]. 

China has more than 1.4 billion people living in a vast territory. “Providing comprehensive lifecycle health services for people" has been the Chinese government’s goal for a very long time. There is no doubt that China has achieved great successes in people’s health and life expectancy since implementing the reform and opening-up policy in 1978. With the rapid development of reform and opening-up, China’s healthcare system has transformed from a centrally planned system into a market-oriented one, which has changed their health care system dramatically in a short time [11]. At the health service providing level, the public hospitals are not the only health service providers; many private hospitals swarmed into the medical service market and become an important component of health service providers. In terms of investment in public health, the government relies on non-government capital to provide public services. As a result of this, the government public–private partnerships mode has gradually prevailed. These changes create a critical underappreciated problem of fragmentation: focusing on the parts without adequately appreciating the whole [12]. The fragmentation is at the heart of inefficiency, disorder, and expensive health systems. Particularly, China’s population is aging at unprecedented levels. The number of people aged 65 and over reached 176 million by the end of 2019 [13]. The fragmented health services system cannot meet the demands of the population, especially the elderly, many of whom often have chronic diseases [14]. 

To solve the most pressing issues, the Communist Party of China (CPC), and the government have increasingly paid attention to changing the fragmentation of health services. Chinese Premier Li Keqiang emphasized that China should launch an integrated care pilot project and strive to provide valuable experience for promotion in the country in 2017. In April of the same year, the General Office of the State Council released a new document named "Guiding Opinions of the General Office of the State Council on Promoting the Construction and Development of integrated care". This document sets specific goals, including building a framework by 2017 and forming systems by 2020. To further promote integrated care development, the Chinese Central Government and Ministries have introduced a series of related policies and regulations to support the development of the integrated care, whose purpose was to improve health service outcomes, quality of life, and patient satisfaction, and reduce costs [1,2]. Integrated care policies include a range of policies that aim to develop integrated care. These policies form a joint force and promote the development of integrated care. With the support of state policies, the reform of integrated care expanded largely and quickly in China. To completely understand the integrated care policies, policy instruments can provide a new perspective. Policy instruments are a range of political actions and behavioral approaches that policymakers have undertaken to achieve a certain policy target [15]. More precisely, the policy instrument is a bridge that can link policy formulation to policy implementation. By analyzing the types and combinations of policy instruments, it is possible to depict the inherent laws within the government’s application of policy instruments. 

It is known that policy instruments have been applied in different fields. However, how the policy instruments would help in promoting the development of integrated care is still relatively little detected by academic analysts. The aim of this paper is to generate fresh insight into this research gap. This paper is organized as follows. Section 2 gives an overview of previous studies on integrated care and policy instruments. Section 3 presents the data sources, analytical framework, the methodology used, and the research process. Section 4 describes the results of the above analysis. Section 5 provides a detailed discussion about the use of policy instruments. Section 6 outlines our main conclusions and suggestions for integrated care. 

## 2. Literature Review

### 2.1. Integrated Care

It is impossible to conduct further study without first addressing the meaning of integrated care. The word integration derived from the Latin verb integer, which means “to complete”. The word ‘integrated’ can be interpreted as ‘reunited parts of a whole’ [16]. From the perspective of systems theory, all organizations have their joint tasks. The completion of joint tasks requires an effective network of division and cooperation. In this sense, integration is similar to a "glue" that connects different elements together; it can utilize resources rationally and achieve the best results. The meaning of integration can help researchers explore the definition of integrated care. However, there are many definitions of integrated care. Of these, some authors, such as Kodner (2011) defined integrated care as a coherent set of methods and models that can integrate the funding, administrative, organizational, and service delivery into a coherent whole [16]. Gillies (2002) defines an organized delivery system as a network that arranges to provide an integrated continuum of services to the population [17]. Sheiman (1994) highlights that teamwork, coordination, and continuity are the key features of integrated care [18]. The terminology of integrated care is used broadly to define a health organization that offers a continuum of healthcare services [6]. As the World Health Organization (WHO) reported, integrated care has widely varying definitions and has no existing unifying definition [19]. In short, the meaning of ‘integrated care’ has multiple meanings depending on the circumstances and period of time [20,21]. However, three typical features can be summarized as following: (i) The goal of integrated care is to provide the adequate service at the right place and the right time; (ii) integrated care is the new health service model, which prioritizes the integration and coordination of services across the spectrum of care, from health promotion and disease prevention to curative and rehabilitation; (iii) integrated care implies a process of rebalancing and structuring the health delivery system into interconnected networks.

According to the review of integrated care, there have been a lot of achievements related to integrated care. Some studies have focused on the assessment of integrated care. For example, Nurjono et al. (2018) use the Rainbow Model of Integrated Care to assess integrated care within a care system as a whole gathered from healthcare providers’ and managerial perspectives [22]. Devers et al. (2016) provide some tested measures based on their research involving nine organized delivery systems. Some researchers examined the key challenge for integrated care systems [23]. Burns (1994) presents some dilemmas which are found in six integrated care systems [24]. Maruthappu (1999) pointed out that operational complexity, regulatory challenges, unclear financial attribution, and cultural inertia have stifled the implementation of integration programs [25]. In addition, Øvretveit (2015) explored how to use digital technologies supporting person-centered integrated care [26]. In short, domestic and overseas scholars have taken up a lot of valuable research on the theory and practice of integrated care. It is worth mentioning that a few researchers focus on integrated care from the perspective of policy instruments. Currently, most of the research on integrated care concentrates on the definition, characteristics, and the assessment. However, the research up to now has not revealed how the concepts of policy instruments can be used to promote the development of integrated care in China; thus, the aim of this research is to generate fresh insight into this research gap.

### 2.2. Policy Instruments

Policy instruments are often known as “governing instruments" or "instruments of government” [27]. As Howlett (1991) said, policy instruments mean the generic term provided to encompass the myriad of techniques at the disposal of governments to implement their public policy objectives [27]. A general and conventional definition of public policy instruments is “a set of techniques by which [the] government [can] make full use their power in attempting to ensure effect and support (or prevent) the changes of whole society” [28]. Although different definitions of policy instruments exist, most of them start from the premise that the government explores policy instruments as an effective method to implement their policies [27,28]. As an important branch of public management, policy instruments have become the focus for many scholars with the development of the new public management movements. Researchers have sharply focused studies on specific types of instruments, such as regulation [29,30], public information campaigns [31], procurement [32], and tax incentives [33,34]. 

Usually, policy instruments may be analyzed as bridge interfaces that connected policy objectives and policy outcomes [35]. In the process of policymaking, the choice of instruments is a crucial decision regarding the formulation of innovation policy. In a way, the implementation of policy target are dependent on the selection of various policy instruments [36]. While implementing policies, the types and the criteria used to evaluate the effectiveness of policy instruments may have a detrimental impact on a government’s ability to achieve its established policy objectives [37]. Therefore, the classification of policy instruments has attracted a great number of scholars’ attention.

Different scholars classified the policy instruments into different types from different angles. Elliott and Salamon (2002) discussed the main government instruments currently in use and summarized their basic characteristics and application patterns in a book called *The Tools of Government: A guide to the New Governance* [38]. Hood’s categorization of policy instruments is resource-based, which includes eight policy instruments, namely, advice, grants/loans, laws, service delivery, surveys, consultants, registration, and statistics [27]. According to Chen’s research, policy instruments contain market instruments, business technologies, and social measures [39]. Howlett and Ramesh (1995) recommend that policy instruments be regarded in terms of a spectrum based on the degree of state involvement in offering the public goods and public services associated with each policy instrument. At the high end of the governmental involvement arc “compulsory” policy instruments, at the low end arc “voluntary” policy instruments, and between the two lie “mixed” policy instruments [40]. Bemelmans et al. (1998) suggested that policy instruments can be divided into regulatory instruments, economic and financial instruments, and soft instruments [28]. Rothwell and Zegveld classify the related policy instruments into three types: the environmental type, the supply type, and the demand type in the light of the impact of policies work in technology [41]. In academic circles, Rothwell and Zegveld’s division remains the most accepted in the literature on instruments, and it continues to be the most widely used in practical contexts. For example, Zhang et al. (2019) analyzed a number of talent policies using Rothwell and Zegveld‘s classification framework on policy instruments [42]. This three-fold division also offers a useful framework and laid a solid foundation for our research. 

**Figure 1:**
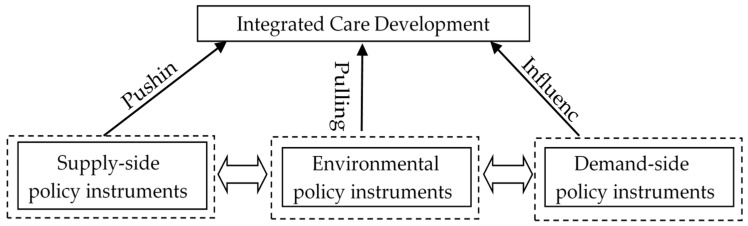
The Policy Instruments for Integrated Care.

Based on Rothwell and Zegveld‘s classification framework, this study classifies integrated care policies into supply-side policy instruments, demand-side policy instruments, and environmental policy instruments. The analysis framework for policy instruments is shown in Figure 1. The demand-side policy instruments mainly play a role in the market, and their significance lies in providing a stable market of integrated care health service through government intervention (e.g., government procurement). Environmental policy instruments are reflected in providing a good environment for the development of integrated care. Supply-side policy instruments are designed to make aggregate supply more responsive to meet people’s ever-growing health needs. Supply-side policy instruments are normally focused on providing support for the development of integrated care, such as removing market barriers to the smooth delivery of health services. In short, supply and demand-type integrated care policies primarily play a direct push or pull role, whereas the environmental type of policy plays an influential role. 

## 3. Methods

**Figure 2:**
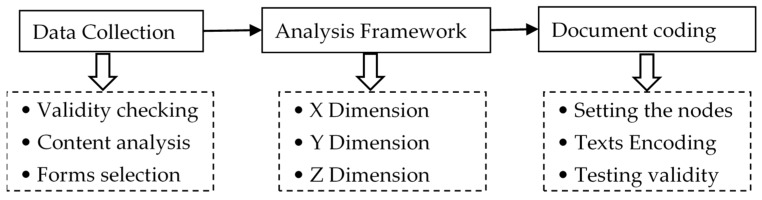
The analysis process for integrated care policy documents.

This study adopts a qualitative document analysis (QDA) method. The QDA is a research method for rigorously and systematically analyzing the contents of written documents [43]. The QDA process entailed the following key three steps, including (a) date collection; (b) setting an analysis framework; and (3) document coding. An overview of what each of these steps entails is show in Figure 2.

### 3.1. Data Collection

Policy documents are the carriers of policy instruments. To obtain comprehensive and representative policy documents for China’s integrated care, this paper selects the documents from the website issued by the central government and various ministries and commissions, including the State Council, the National Health Commission, the National Development and Reform Commission, the Ministry of Human Resources and Social Security, the National Development and Reform Commission, the National Administration Traditional Chinese Medicine, and other relevant ministries and commissions. It is worth noting that the integrated care policies issued by local authorities are excluded. Seventy-five relevant documents were initially collected. To ensure reliability and validity, this study took the following three principles to further screen the selected policy documents.

1. Validity checking. Invalid policies are meaningless to our research. With the advance of health reform, some of the health policies are being dismantled. The authors firstly checked the validity of the collected policies. As a result, five invalid outdated policies were abandoned.

2. Content analysis. As Glenn (2009) has suggested, content analysis involves thinking about whether and how documents can serve the research purposes [44]. To serve the research aim, the content of policy documents was selected as an important reference index for us to select policy texts. Each document was analyzed to determine the extent to which the policy described or addressed the integrated care health service issue. After reading all the contents of the collected policy documents carefully, the authors selected 43 documents that explicitly targeted integrated care. 

3. Form selection. The forms of policies chosen were mainly laws, strategies, regulations, plans, opinions, guiding opinions, measures, announcements, and notices that directly reflect the government’s attitude toward integrated care; reply, approval, and industry standards were not included.

With the expectation of achieving higher research quality, all of the authors in this paper participated in the process of policy screening. Finally, 43 policy documents associated with integrated care policies ultimately met the selection criteria and were selected as analyzed policy databases.

### 3.2. An Analytical Framework

#### 3.2.1. X Dimension: The Basic Policy Instrument

Combining Rothwell and Zegveld’s classification with the content of China’s integrated care policies [41], this paper divides integrated care policy instruments into three types. Table 1 presents the frame of policy instruments for China’s integrated care development. The demand-side policy instruments have a pulling role in the further development of integrated care. It includes four sub- policy instruments, namely, family doctor engagement, Medicare reimbursement, government procurement, and private sector support. Supply-side policy instruments play a pushing role in the development of integrated care. According to the implementation of China’s integrated care policies, supply-side policy instruments are segmented into talent support, technology information support, capital investment, and infrastructure construction. Environmental policy instruments can influence the development of integrated care and are classified into target planning, performance appraisal and rewards, regulation control, and policy advocacy.

#### 3.2.2. Y Dimension: Stakeholders 

An integrated care system is a complex services network that links many stakeholders [45,46]. The existing tiered healthcare system involves multiple parties, such as the demand, supply, management, financing sides, and other parties [47]. Identifying the various key stakeholders and regulating their behaviors using policy instruments is particularly crucial to promote the effectiveness of integrated care.

**Figure 3:**
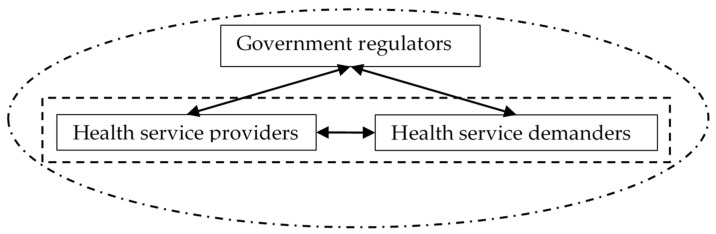
The stakeholders of integrated care.

The study classified stakeholders into three categories: the regulators (the government), the provider providers (medical institutions), and the demanders (the public), as can be seen in Figure 3. The members of the health service providers in the integrated care system mainly include various medical institutions that directly provide medical services for people. In China, there are three levels of hospitals: first-level hospitals provide basic health care services to a community territory or an organization; second-level hospitals are comprehensive hospitals with specialist services but are relatively small scale; third-level hospitals are large-scale comprehensive hospitals. The health service demander mainly refers to patients and potential health service users. The regulator mainly includes central ministries and commissions. For example, the State Council has overall responsibility for national health legislation, policy, and administration. The National Health Commission (NHC) takes the responsibility for the formulation of health policy, ordinating the different levels of health services, and overseeing the health services market [48]. The National Health Insurance Agency (NHIA) is primarily responsible for supervising the operation of the insurance fund, purchasing drugs, and pushing ahead the healthcare reform. The Ministry of Finance (MOF) is engaging in budget preparation for health care development. The Ministry of Human Resource and Social Security (MOHRSS) is responsible for national labor regulations and managing the national social security. The National Administration of Traditional Chinese Medicine (NATCM) is in charge of the regulation of the traditional Chinese medicine industry.

#### 3.2.3. Z Dimension: Health Service Supply Chains

The aim of integrated care is to provide comprehensive health services for the general public. It is an approach that consists of health promotion, disease prevention, and curative services [49]. Generally, comprehensive health services include three major parts—prevention, treatment, and rehabilitation—which can be regarded as the important parts of healthcare [50,51]. Similar to supply chain management in a manufacturing setting [52], effective management in health service supply chains can achieve substantial savings and better focus on their core patient care mission [53]. 

Prevention, as it relates to health, is generally about avoiding disease before it starts. It has been defined as the plans for, and the measures taken, to prevent the onset of a disease or other health problems before the occurrence of the undesirable health event. Treatment are the manners in which a disease is cared for or dealt with. Rehabilitation is a process that can help you keep or improve abilities that you need for daily life. These abilities may be physical, mental, and/or cognitive (thinking and learning). These three supply chains attach closely to each other. 

**Figure 4:**
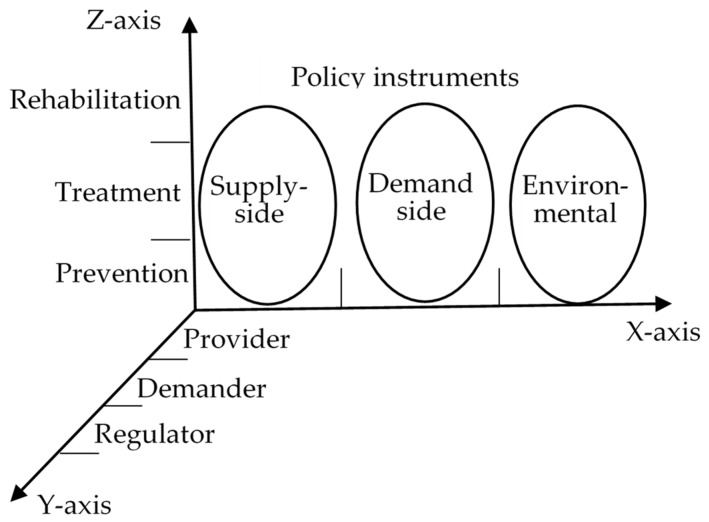
Three-dimensional analytical framework for integrated care policies.

Aiming at conducting comprehensive policy analysis, this study designs a whole framework for an X-Y-Z visualization of integrated care policies analysis, as shown in Figure 4. The framework draws on Rothwell and Zegveld‘s classification framework of policy instruments and adds two new dimensions—the stakeholder dimension and health service supply chains dimension—to support policy analysis. The framework offers a reference model for researchers to conduct policy text analysis and lays a foundation for policymakers to further improve integrated care policies in China.

### 3.3. Document Coding Process

Coding is a ubiquitous part of the qualitative document analysis. Usually, words, phrases, and sentences were defined as the unit of observation and classified to different types of policy instruments in the process of coding. Therefore, the policy instruments can be revealed. The process of document coding includes the following steps.

1. Setting the nodes. A complete understanding of the research topic is essential for researchers before they start to set the nodes. Table 1 shows the types of policy instruments, which laid the foundation for setting the nodes. Setting the nodes is a typical top–down approach, in which researchers put the encoded content into the predetermined node. Usually, the predetermined nodes include tree nodes and subpoint nodes. This research sets the policy instrument type as the tree nodes and then sets the policy instrument sub-type as the subpoint nodes. 

2. Texts Encoding. Coding is an effective way of gathering all the corresponding reference points to a specific policy instrument. In order to improve the accuracy of analysis, we use QSR NVIVO 11 software as an assist tool to conduct a content analysis of policy texts [54]. NVivo is a piece of qualitative analysis software that is widely used by academic, government, and commercial health researchers across a diverse range of fields. Its powerful coding, query, and classification functions can help us conveniently analyze public policies.

3. Testing validity. To ensure the consistency and reliability of the coding, the analysis of every document was verified by two researchers who independently screened the integrated care policies according to coding criteria. When they arrived at different points, the researchers always stopped to discuss and reach an agreement before going on to the next step. To ensure the validity, a third person also served as an arbiter for any inconsistencies between the two primary coders and ensured robust interpretative analysis and conclusions.

## 4. Results

### 4.1. Overall Analysis of Policy Instruments

The number of coded references can describe the using frequency of policy instruments. We queried the coded references of every node and calculate the proportion of different policy instrument in the total policy document; the results are shown in Table 2. It clearly shows that the coded references among different sub-policy instruments are different. Specifically, the supply-side policy instruments focused mostly on talent support (36 items in total), technology information support (38 items in total), and capital investment (29 items in total). There are 17 items related to infrastructure construction.

Among the demand-side policy instruments, the frequency of Medicare reimbursement is the highest, with a total of 27. The usage frequencies of government procurement, social sector support, and family doctor engagement are respectively 16, 24, and 13.

Through further analysis of the environmental policy instruments, the regulation control is the most frequently used in policy instruments, with a total of 48 items. The usage frequencies of the target planning, performance appraisal and rewards, and policy advocacy are respectively 22, 38, and 31.

**Figure 5:**
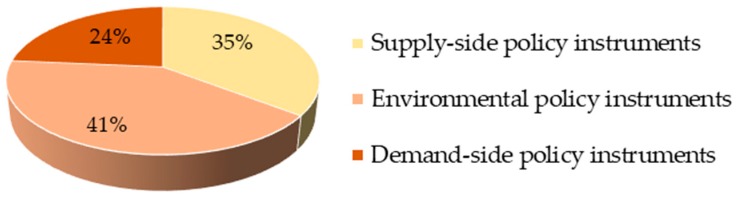
Policy instruments for integrated care.

The coded references of sub-policy instruments determine the proportion of different policy instruments. Overall, the environmental policy instruments accounts for 41%. The next largest policy instruments are supply-side policy instruments (35%), while the demand-side policy instruments account for the smallest percent of policy instruments (24%). The results are shown in Figure 5. 

### 4.2. Policy Instruments Used in Stakeholders Dimension

The X–Y dimension focuses on policy instruments from the stakeholder perspective. As mentioned above, the stakeholders mainly include providers, regulators, and demanders. The proportion of these three types of policy instruments used in stakeholders is shown in Table 3. On a whole, the number of policy instruments related to the providers rank in the first place (98 items in total). This is followed by the policy instruments related to the regulators (67 items in total). The number of policy instruments related to the demanders ranks last (64 items in total).

### 4.3. Policy Instruments Used in Health Service Supply Chains Dimention

**Figure 6:**
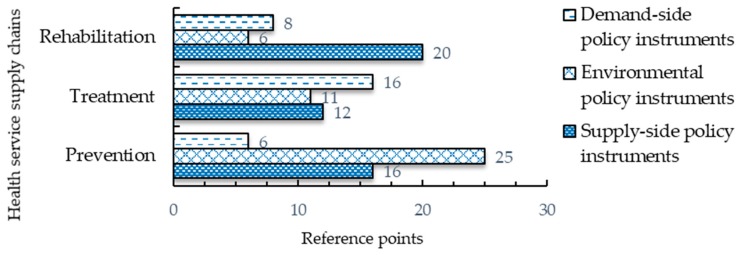
Frequency distribution of policy instruments in different stages of health service supply chains.

The X–Z dimension focuses on analyzing policy instruments from the health service supply chains’ perspective. The health service supply chains include basic three links: prevention, treatment, and rehabilitation. The frequencies of the policy instruments used in these three links are shown in Figure 6. All in all, the number of policy instruments for prevention ranks in the first place (47 items in total). Goal-planning instruments were adopted most frequently, followed by regulation control instruments. This is followed by the policy instruments for treatment (39 items in total). The number of policy instruments for rehabilitation is last (34 items in total). 

## 5. Discussion 

The results summarized above show that some policy instruments have the potential to contribute to the development of integrated care, whereas other show a low potential or need for redesign. This section aims to put stress on exploring the potential reasonable explanation for the use of policy instruments and discuss whether any adjustments to policy instruments are needed.

### 5.1. The Using of Supply-Side Policy Instruments

Talent support is a major undertaking of Chinese medical service reform. Doctors play a key role in patient treatment and recovery. To improve the quality of medical services, the Chinese government departments formulate long-term talent development policies that include establishing the system of training and developing the education of general practitioners. Technology information support can improve the efficiency of health services. Internet medicine is regarded as a way of creating a more sustainable system [55]. The Chinese government has attached great importance to the application and development of digital health and care. The whole country is going to great lengths to establish a regional platform of the health information system (HIS). New telecollaboration networks of the “Internet medical and health” field have emerged. For example, China–Japan Friendship Hospital can provide telemedicine, teaching, and training services for remote and underdeveloped areas. Financial support for health care is the responsibility of the government. From a historical point of view, China has made dramatic progress in the health care system. 

However, hospital dominance in health service delivery has not changed. In fact, primary healthcare’s role is limited. Since a new round of health reforms was launched in 2009, China’s central government continues to support the development of grassroots medical institutions and perfect the mechanism of public health budget. To enhance the construction of basic infrastructure is a significant foundation for planning, delivering, evaluating, and improving public health. The actions of strengthening infrastructure development mainly include three parts: establishing a network of public sports facilities, upgrading the hardware facilities of primary healthcare, and strengthening the medical staff training center construction.

### 5.2. The Using of Demand-Side Policy Instruments

The effect of demand-side policy instruments is more obvious than the other two types of policy instruments. Medicare reimbursement is the basic and most used among the demand-side policy instruments, giving full play to the Medicare reimbursement policy instrument. The medical insurance policy instrument is mainly used in health organizations and for patients. For health organizations, new payment methods, such as the diagnosis-related group (DRG) payment system, have emerged. For ordinary patients, the reimbursement rates of grassroots medical institutions should be substantially increased from the existing basis and the gap of reimbursement rates between tertiary hospitals and grassroots medical institutions has widened further [56]. China adopted a family doctor policy to provide basic healthcare to the whole population. It is expected that family doctors who serve as health gatekeepers for residents will help solve health challenges such as the increasing number of patients with chronic diseases. The implementation of a family doctor policy can alleviate the crowding in the largest hospitals. Improving the professional skills and raising the wage of family doctors are the focus of current policies. Government procurement refers to a government’s purchase of services from grassroots medical institutions run by social organizations or a private person. In recent decades, the Chinese government has attempted to innovative public health services and expand the scope of government procurement. For instance, the government shall grant to the private hospitals or private clinics social security-designated organizations that can meet the requirements of government. Social sectors are important participators of integrated care development. In China, the government has taken some effective measures to encourage social forces and capital investment in health service. As a result, the number of the civilian-run hospitals has exceeded that of public hospitals. The rapid development of civilian-run hospitals can meet the multi-level and diversified demand of medical services to some degree.

### 5.3. The Use of Environmental Policy Instruments 

On average, the number of environmental policy instruments is higher than those of other instruments. In particularly, various sub-instruments of environmental policy instruments have different functions. Target planning is drawn up to accomplish desirable ends, which is an essential step for policy implementation. The construction of integrated care is expected to proceed in two phases: the first phase is to set up the institutional framework to provide the basic platform for integrated care development. In the second, integrated care development should be advanced in all respects, and a perfect policy supporting system should be formed according to valuable pilot experience. Performance appraisal and rewards give recognition or rewards to employees or organizations whose work advances the expected goals. The appraisal indicators system has been issued by the central government in order to make the integrated care performance appraisal system more scientific and systematic. The wage and incentive systems of integrated care must be in conformity with the principle of distribution according to work and more pay for more work. To activate integration, certain rules and regulations are required. The results showed that the number of regulation control instruments is larger than that of others. Regulation control instruments are mainly used for setting prices for medical services, ensuring the quality of medical service, and strengthening the quality of drug safety supervision. Usually, the regulation control instruments can often yield immediate results, but its effect is often strongly associated with the strength of policy enforcement. To create a sound environment for integrated care development and respond to people’s concerns promptly, the government has attached importance to publicize integrated care development through various channels. For instance, the government holds press conferences to give the "integrated care" policy interpretation, including the basic content, objectives, and implementation of the principles and implementation mechanisms. 

### 5.4. The Use of Policy Instruments by Different Stakeholders

The development of integrated care involves multiple stakeholders. Among stakeholders, regulators, demanders, and providers are recognized as the three important participants. In the process of integrated care development, the government should play a good role as regulators, because governments around the world should fulfill their responsibilities to provide good medical services for their people. Health information asymmetry and the disorder of the market need to fully play the role of administrative supervision. Therefore, regulation control is the most frequently used policy instrument in practice. The regulation is mainly used for the quality and safety of health service control and the social medical insurance fund supervision. Meanwhile, regulation control was also the main policy instrument used for providers. Establishing effective and regulated operations of the healthcare system requires regulation control to reduce the blindness of the market mechanism. In addition, performance appraisal and rewards are the most commonly used forms of regulation control for providers. 

Demanders, as one of the most active types of participants in health service, are often seen as passive recipients in the medical services process. Comparing regulators and providers, demanders have received less attention from policy instruments. The policy instruments used for demanders are mainly policy advocacy. The aim of policy advocacy is to motivate healthy lifestyles at all life stages for people and to strengthen health promotion and education and enhance the health awareness and the self-care ability of the people.

### 5.5. The Using of Policy Instruments in Health Service Supply Chains

The major roadblock of integrated care development is the fragmentation of health service delivery, which caused the breaking of the health service chain over the last decades. Prevention, treatment, and rehabilitation are the three essential parts of the health service supply chains. 

The results of this study show that prevention has become the focus of policy instruments. With the changes of dietary patterns, lifestyles, and the disease patterns among the Chinese population, disease prevention and control have become the most pressing concerns. That is the reason why policy instruments focus on prevention. Treatment, as important link in health service, is still the focus of policy instruments. However, rehabilitation services have received limited attention. Integrated care is accepted as a complicated process; hence, the systematic view is strongly advocated [57]. The combination of prevention, treatment, and rehabilitation is the pressing task for the development of integrated care.

### 5.6. Limitation and Further Research Directions

Nevertheless, this paper has some limitations. Firstly, this paper only explores how policy instruments promote the development of integrated care. However, this study has filled the gap of current studies to some degree. It is acknowledged that the development of integrated care is affected by many factors. In practice, the role of policy instruments has an important role in the development of integrated care, but they cannot directly determine the development of integrated care. Secondly, the conclusion of this paper is limited by the classification of policy instruments. Admittedly, there are alternative classifications of policy instruments [28,58].

Due to the complexity of policy instruments, a complete in-depth analysis of policy instruments for integrated care is not easily carry out. What kind of classification method is adopted has a direct impact on the research conclusion. This study adopted a threefold classification of policy instruments, which leads to a framework for research results. Thirdly, the content analysis method is a common research method. Although we systematically coded the policy documents based strictly on established criteria, it is still unavoidably affected by the cultural knowledge and cultural background of the researcher.

However, this study provides insight into understanding the policy instruments used for integrated care in China. The research findings can extend our knowledge about the use of policy instruments for integrated care. The results also help the policymaker gain a better understanding of integrated care policies, which can provide a practical guidance to the development of integrated care. Future work might focus on the practice of integrated care in China to explore the effectiveness of policy instruments.

## 6. Conclusions and Suggestions

### 6.1. Conclusions

Providing integrated care that crosses the boundaries between grassroots medical institutions, secondary hospitals, and tertiary hospitals is the goal of many countries worldwide. The policy instruments are a bridge between the goal and reality. An in-depth analysis of the relevant policy instruments used by the central government will help the policy implementer fully understand the blueprint planning of integrated care and accelerate the pace of implementation of integrated care. Using the content analysis method, this paper explores the using of policy instruments through examining a range of policies on integrated care from 2009 to 2019. The findings of this study are as follows.

Firstly, supply-side policy instruments are a way to meet the public’s diversified demands. The demand-side policy practices related to integrated care in China cover a range of policy instruments, including talent support, technology information support, capital investment, and infrastructure construction. The results show that the government highlights preferential policies to explore personnel training. “Talent support” policy instruments have experienced high-frequency use. When comparing the environmental and supply types of policy instruments, China has adopted relatively fewer demand-side policy instruments. This indicates that demand-side policy instruments have not received more attention, although they play an indispensable role in the development of integrated care. The inadequate application of demand-side policy instruments reduces their instrumental value in balancing demand and supply. In demand-side policy instruments, Medicare reimbursement has the highest proportion, which reflects the intention of how the government begins to explore various forms of payment of medical insurance fund. The new system of payment for Medicare reimbursement is still probed actively. Relatively speaking, the specificity and the clarity of demand-side policy instruments are weak. The proportions of environmental policy instruments have risen to 40%, reflecting the government’s intention to create a sustainable environment for integrated care development by macro-policy. This type of policy instrument can indirectly influence the development of integrated care. It should be remarkable that target planning sub-policy instruments are used by the government to guide the actions of policy-related organizations. To some extent, target planning can be interpreted as policy targets. Among the environmental policy instruments, “administrative regulation” is used more frequently.

Additionally, the development of integrated care needs to fully address the roles of providers, demanders, and regulators. The government places more emphasis on providers, followed by regulators. One of the key achievements of successful integrated care was the clear expectations for medical institutions’ functions, responsibilities, and accountabilities. Vital prerequisites for coordinated service delivery include an agreement regarding who does what, when, and where, and how information is communicated, naming responsibilities and clarifying tasks. As the regulator, the government has increased the investment of public health infrastructure and strengthened the full monitoring of financial funds to improve the efficiency of money utilization. The frequency of policy instruments used for the demanders is the least. The demanders also should be encouraged to participate in integrated care undertakings.

Furthermore, the results show that the policy instruments had been used for the whole health service chains. The different stages of health service chains have attracted different amounts of attention. The prevention stage has received more attention. Meanwhile, less attention has been paid to the rehabilitation stage. Since the implementation of health care reforms, much has been documented regarding the practices and behavior of integrated care in China. While these documentations pointed to various problems arising from the reforms, a systematic analysis focused on policy instruments is rarely found. The study contributes new evidence regarding the development of integrated care, with particular emphasis on the use of policy instruments. Through comparing our findings to the findings of earlier studies, the precise way in which China has approached integrated care through policy instruments is revealed. This study presents a solid and operational framework for filling existing gaps in knowledge about policy instruments used for the development of integrated care in China.

### 6.2. Policy Recommendations

Based on the detailed policy instruments analysis above, this paper proposes the following policy recommendations, which may optimize integrated care policy instruments and might provide a useful reference for policymakers.

Firstly, the government should improve the efficiency of the “policy instruments mix”. China’s integrated care policies mainly adopt the environmental policy instruments, followed by supply-side policy instruments, and a few demand-side policy instruments have been proposed. The effect of policy implementation is affected by the combination of policy instruments. All instruments have strengths and weaknesses. The policy instruments should not be regarded or implemented as independent sets. Their rollout will require synchronization [59]. To address this situation, the government should focus on the demand policy instruments, supplement supply policy instruments, and develop environmental instruments to achieve a combination of policy instruments. In short, the three types of policy instruments represent a comprehensive package of interventions to deepen integrated care reform. 

Additionally, the government should optimize the policy instruments according to the trend of social development. The selection of policy instruments should fully consider not only the process of integrated care reform but also the social contexts [60]. At present, the rapid development of the Internet has created new opportunities for health reform. The Chinese government has attached importance to Internet healthcare. However, most of the existing policies only provide guidance for the development of Internet healthcare at the macro level. The specific operating measures are almost absent, which reduces the practical feasibility of policy instruments. Therefore, it is necessary to clarify the detailed development strategy, such as protecting patient privacy, monitoring and managing the hospitals and doctors, and carrying firm punishments for illegal operation. Under the current circumstances, the government should adopt all kinds of policy instruments to support the development of Internet healthcare.

Furthermore, the government should pay extensive attention to the demanders. The implementing of integrated care policies is not a purely ‘technical’ challenge but rather one that involves a great deal of stakeholders. The development of integrated care depends on the success of trilateral cooperation among the regulators (the government), providers (medical institutions), and demanders (the public). The policy instruments related to providers and regulators are relatively abundant, whereas the demanders get less attention. Demanders are as important as the other participants, because they can directly influence policy implementation. The Chinese government claims that China’s health policy is primarily focused on patient-oriented ideas. Yet, the emphasis on health service demanders is far from enough. In practice, as the demanders, the public has not played their role in expressing their needs directly. Consequently, it is necessary to set up a perfect multiple participation mechanism to promote integrated care development. Specifically speaking, the intensity of the use of social sector support policy instruments can be strengthened to involve the entire society in supporting the development of integrated care. The Chinese government should speed up the establishment of a social participation mechanism for the development of integrated care. In addition, the government must continue to strengthen public education on the healthcare consciousness and increase the public participation in integrated care governance. In addition, public health education should also be recognized as an important acting strategy to enhance the public healthcare consciousness. Demanders, as well as providers and regulators, should also be the focus of the integrated care development.

Lastly, we need to put the prevention concept into practice. It is a long journey to cure disease. Prevention is far better than cure. The results have shown that China’s integrated care policy is primarily focused on prevention. However, in practice, this does not appear to be the case [23]. In order to further improve the level of prevention, the government should play the full role of the family doctor in recognizing important diseases and managing most acute and chronic illnesses [61]. The role of family doctors has proven to be effective for chronic disease prevention in many high-income countries. In China, the developmental space of family doctors is still very large. In addition, the policy instruments should put the emphasis on improving the existing self-care and cultivating healthy lifestyles. 

## Figures and Tables

**Table 1:** The classification of policy instruments.

Policy type	Instrument name	Description
Supply-side policy instruments	Talent support (TS)	Formulating long-term talent development policies for promoting integrated care development, which include increasing the number of primary care practitioners and improving the skills of medical staff.
	Technology information support (TIS)	Providing public scientific and technological support and information services for integrated care development.
	Capital investment (CI)	The government directly provides financial resources for integrated care development.
	Infrastructure construction (IC)	Updating basic public health infrastructures and expanding health service facilities.
Demand-side policy instruments	Family doctor engagement (FDE)	Providing basic healthcare to the whole population, helping manage health and medical costs.
	Medicare reimbursement (MR)	Expanding the insurance coverage and widening the gap of reimbursement rates among different institutions.
	Government procurement (GP)	Priority to purchase basic public health services and centralized drug purchasing with public funds is provided by public institutions and organizations.
	Social sector support (SSS)	Encouraging qualified private actors and social organizations to provide or serve the health services.
Environmental policy instruments	Target planning (TP)	A component of the integrated care policies by setting a timetable and determining a plan to achieve the policy goal.
	Performance appraisal and rewards (PAR)	Evaluating achievements and rewarding for high performance.
	Regulation control (RC)	The government enacts a series of laws and regulations to restrict or maintain health services market behavior and to create a favorable environment for people.
	Policy advocacy (PA)	The government publicize integrated care policies and improve the cognition of the masses through various channels and in various ways.

**Table 2:** Coded references statistics.

Node	Sub-Node/Instrument Name	Coded References	Percentage
Supply-side policy instruments	Talent support (TS)	36	10.6%
	Technology information support (TIS)	38	11.2%
	Capital investment (CI)	29	8.6%
	Infrastructure construction (IC)	17	5.0%
Demand-side policy instruments	Family doctor engagement (FDE)	13	3.8%
	Medicare reimbursement (MR)	27	8.0%
	Government procurement (GP)	16	4.7%
	Social sector support (SSS)	24	7.1%
Environmental policy instruments	Target planning (TP)	22	6.5%
	Performance appraisal and rewards (PAR)	38	11.2%
	Regulation control (RC)	48	14.2%
	Policy advocacy (PA)	31	9.1%

**Table 3:** Coded references of policy instruments based on stakeholders.

Stakeholders	Policy Instruments	Coded References	Total Coded References
Demanders	Supply-side policy instruments	39	64
	Environmental policy instruments	45	
	Demand-side policy instruments	14	
Providers	Supply-side policy instruments	39	98
	Environmental policy instruments	45	
	Demand-side policy instruments	14	
Regulators	Supply-side policy instruments	15	67
	Environmental policy instruments	37	
	Demand-side policy instruments	15	
